# Unexpected Hair Regrowth Following Oral Bicalutamide Exposure in a Male Patient With Androgenetic Alopecia and a Suboptimal Response to Conventional Therapies: A Case Report

**DOI:** 10.7759/cureus.112176

**Published:** 2026-07-06

**Authors:** Jared Martínez Coronado, Kenia Dominguez Romo, Luisa F Vazquez Enriquez, Jose Hurtado Cordova, Carolina Pico

**Affiliations:** 1 Department of Dermatology, Skin Hospital, Saltillo, MEX; 2 Department of Internal Medicine, Hospital General de Zona No. 33, Instituto Mexicano del Seguro Social, Monterrey, MEX

**Keywords:** androgenetic alopecia, androgen receptor blockade, bicalutamide, drug adverse effects, hair regrowth, male pattern hair loss, off-label therapy, treatment-resistant alopecia, trichology

## Abstract

Androgenetic alopecia (AGA) is the most common cause of non-scarring hair loss in men. Although topical minoxidil and oral finasteride are established treatments, some patients show limited clinical response despite prolonged therapy. Evidence on the use of oral bicalutamide in male AGA remains scarce because of concerns about systemic adverse effects. We report the case of a 45-year-old man with treatment-resistant AGA who experienced clinically apparent hair regrowth during oral bicalutamide exposure after an inadequate response to conventional therapies. Treatment was accompanied by gynecomastia, mastodynia, decreased libido, erectile dysfunction, and mild elevations in hepatic enzymes, leading to drug discontinuation. Clinical adverse effects resolved after withdrawal of bicalutamide; however, follow-up hormonal evaluation and liver function tests were unavailable. This case describes a temporal association between androgen receptor blockade, clinically apparent hair regrowth, and clinically significant adverse effects. Although the findings provide additional insight into the role of androgen receptor signaling in AGA, they should not be interpreted as evidence supporting the use of oral bicalutamide in male patients. Further controlled studies are needed to better define the safety profile and potential role of androgen receptor antagonism in androgenetic alopecia.

## Introduction

Androgenetic alopecia (AGA) is the most common cause of non-scarring hair loss worldwide and affects a substantial proportion of men throughout their lifetime [1]. The condition is characterized by progressive follicular miniaturization driven by androgen signaling in genetically predisposed individuals, resulting in decreased hair density and patterned hair loss [1]. Beyond its cosmetic manifestations, AGA may have a considerable negative impact on self-esteem, body image, and quality of life.

Current treatment options for male AGA primarily include topical minoxidil and oral finasteride, both of which have demonstrated efficacy in slowing disease progression and promoting hair growth [1]. Nevertheless, some patients continue to experience progressive hair loss or only limited clinical improvement despite prolonged therapy, highlighting the need for a better understanding of treatment responses and the biological pathways involved in disease progression.

Bicalutamide is a nonsteroidal selective androgen receptor antagonist originally developed for the treatment of prostate cancer. Unlike 5-alpha reductase inhibitors, which inhibit the conversion of testosterone to dihydrotestosterone (DHT), bicalutamide competitively antagonizes androgen receptors at the tissue level. Because androgen receptor signaling plays a central role in the pathogenesis of AGA, receptor antagonism has generated interest as a potential area of investigation. More recently, bicalutamide has been evaluated as an off-label treatment for female pattern hair loss, particularly in women with signs of androgen excess, although the available evidence remains limited [2-5]. In contrast, its use in male patients with AGA remains extremely limited because of concerns about clinically significant adverse effects, including gynecomastia, sexual dysfunction, and hepatotoxicity [5-7]. Consequently, the use of oral bicalutamide in male AGA is not established, and its safety profile remains an important concern.

We report the case of a man with long-standing AGA and a suboptimal response to conventional therapy who developed clinically apparent hair regrowth during oral bicalutamide exposure but also experienced endocrine, sexual, and hepatic adverse effects that led to treatment discontinuation. This case is presented to describe this temporal association while emphasizing the importance of carefully balancing any biological observations against the potential risks associated with systemic androgen receptor blockade in male patients.

## Case presentation

A 45-year-old man with no significant past medical history presented to the dermatology clinic with progressive hair loss of approximately 15 years' duration.

**Figure 1:**
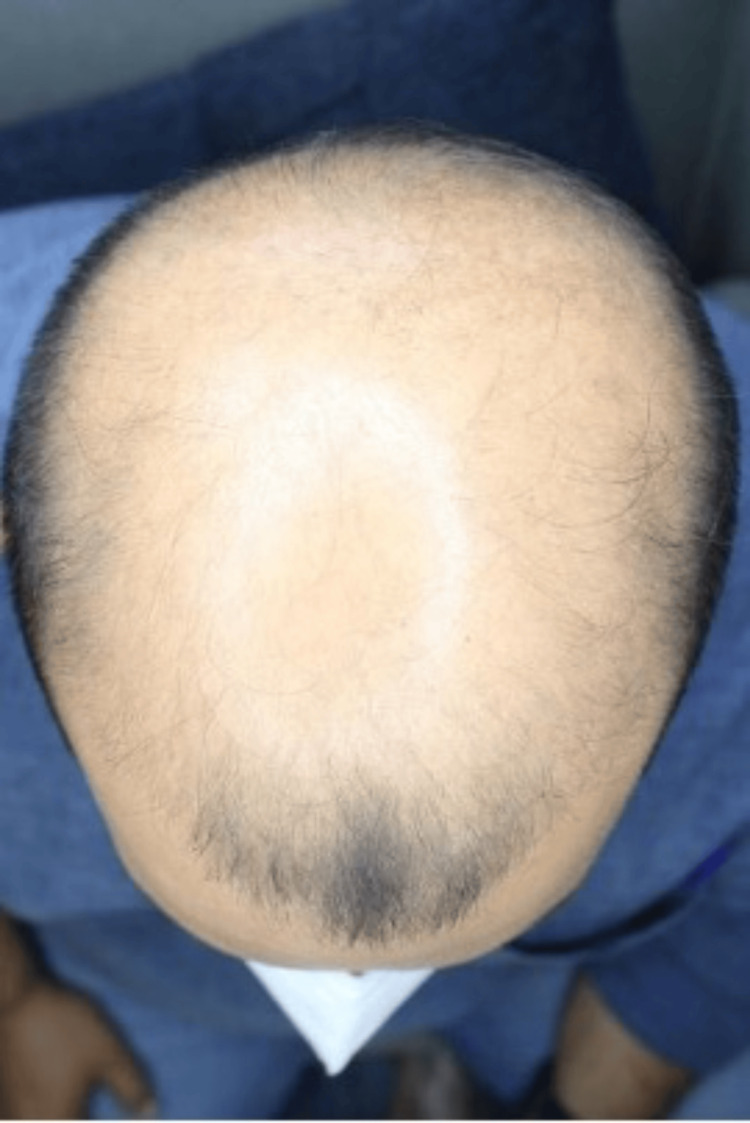
Baseline clinical appearance of the scalp Marked diffuse thinning involving the frontal, mid-scalp, and vertex regions with extensive scalp exposure and preservation of the occipital hair-bearing area.

Physical examination revealed a marked reduction in hair density involving the frontal, mid-scalp, and vertex regions (Figure 1). Trichoscopy demonstrated variation in hair shaft diameter, follicular miniaturization, and reduced follicular density, findings consistent with androgenetic alopecia. Based on the clinical and trichoscopic findings, a diagnosis of androgenetic alopecia, Norwood-Hamilton stage VI [8], was established.

**Figure 2:**
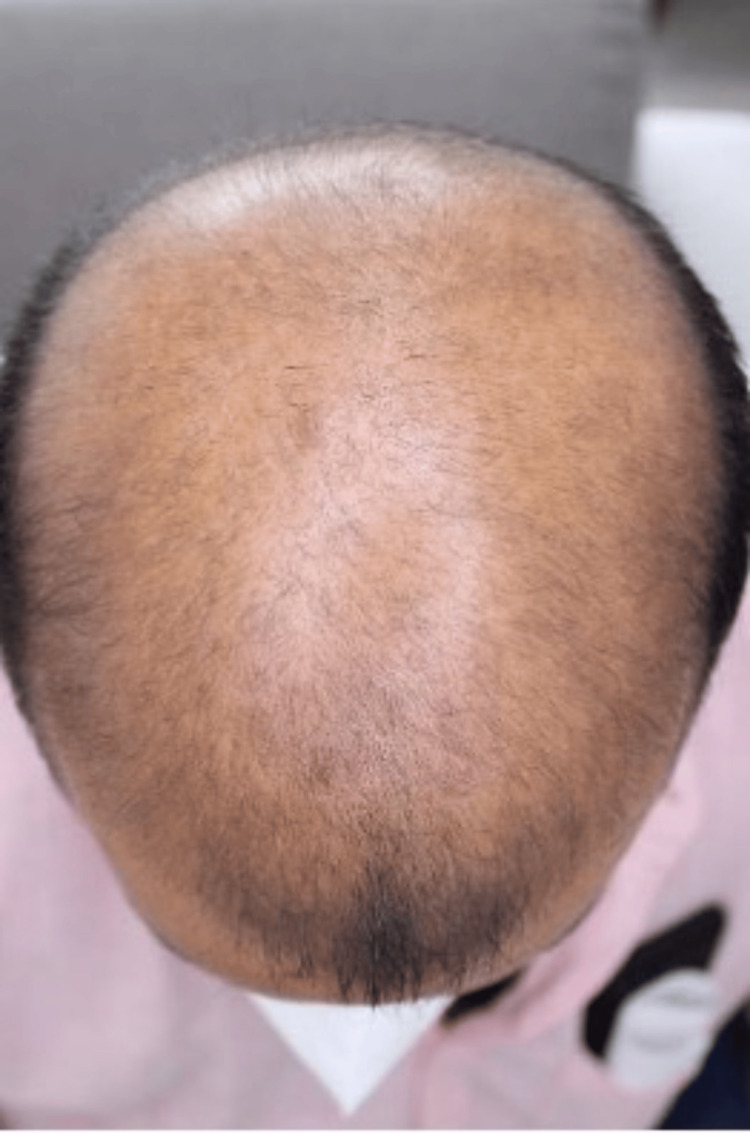
Clinical appearance after approximately two years of conventional therapy Follow-up clinical image demonstrating mild improvement in hair density compared with baseline, with persistent diffuse thinning and scalp visibility involving the frontal, mid-scalp, and vertex regions.

The patient had previously received multiple conventional therapies, including topical minoxidil 5%, oral finasteride 1.25 mg/day, and oral minoxidil 5 mg/day. Despite approximately two years of treatment, only modest clinical improvement was observed, with persistent diffuse thinning and scalp visibility (Figure 2). Because of this suboptimal response to conventional therapy, both topical and oral minoxidil were discontinued before oral bicalutamide 25 mg/day was initiated.

**Figure 3:**
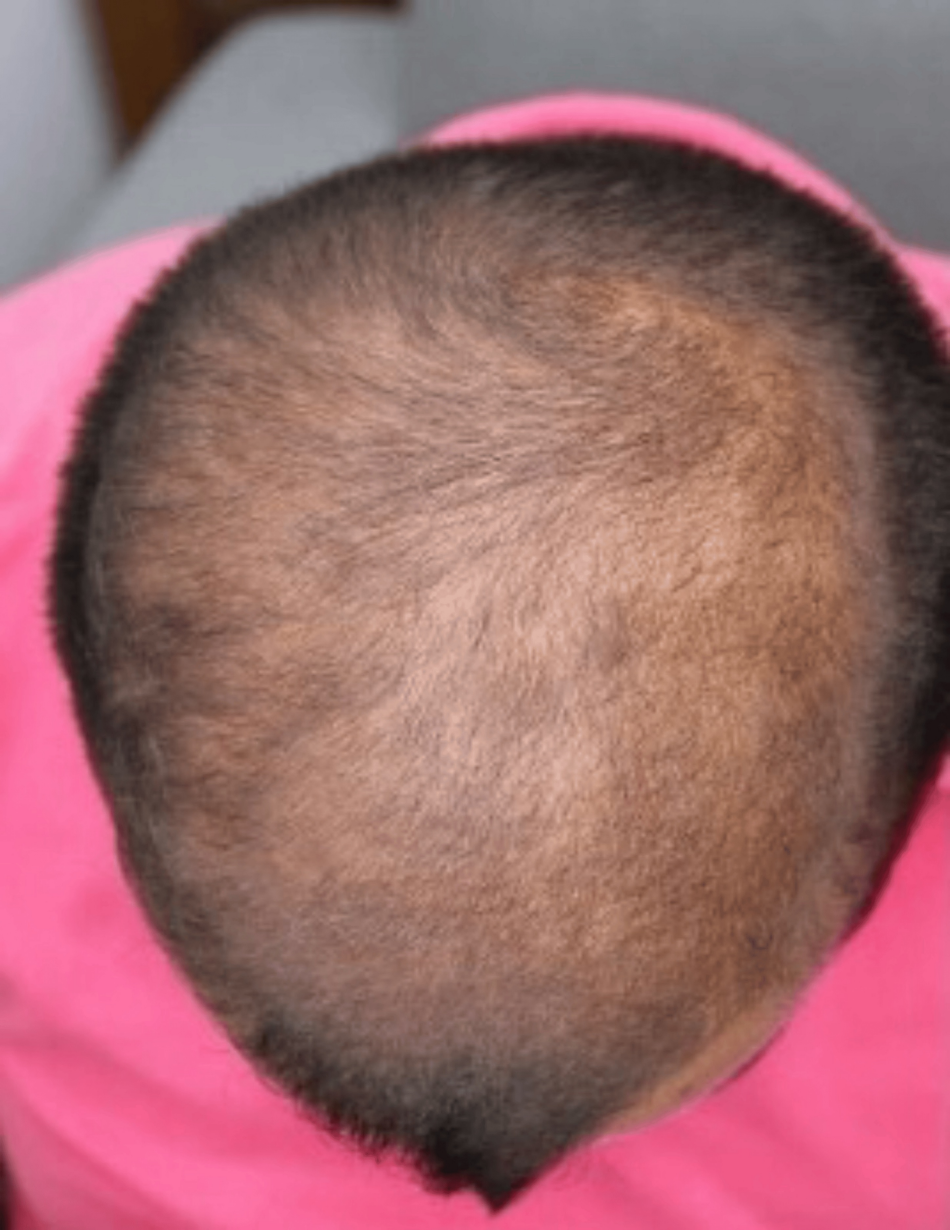
Hair regrowth following bicalutamide exposure Clinical image obtained after four months of treatment with oral bicalutamide 25 mg daily showing noticeable improvement in hair density and scalp coverage compared with baseline and previous follow-up photographs.

Baseline liver function tests obtained before treatment initiation were within normal limits (Table 1). Four months after starting oral bicalutamide, clinically apparent improvement in hair density and scalp coverage, particularly in the crown region, was observed (Figure 3). During the same period, the patient developed gynecomastia, mastodynia, decreased libido, erectile dysfunction, and mild elevations in hepatic enzymes compared with baseline values (Table 1). Hormonal evaluation was not available before, during, or after treatment.

**Table 1:** Liver function tests before initiation of bicalutamide and after four months of treatment Baseline and follow-up liver function tests demonstrating mild elevation of hepatic enzymes during bicalutamide therapy, while bilirubin levels remained within normal limits. AST: aspartate aminotransferase, ALT: alanine aminotransferase, GGT: gamma-glutamyl transferase

Parameter	Reference range	Baseline	Four months after bicalutamide
AST (U/L)	10-40	27	56
ALT (U/L)	7-56	33	78
Alkaline phosphatase (U/L)	44-147	88	96
Total bilirubin (mg/dL)	0.2-1.2	0.8	0.9
GGT (U/L)	8-61	25	48

Because of the development of clinically significant adverse effects, oral bicalutamide was discontinued. Seven weeks after treatment withdrawal, gynecomastia, mastodynia, decreased libido, and erectile dysfunction had completely resolved. Follow-up liver function tests were not available. The patient subsequently resumed treatment with oral minoxidil 5 mg/day and a topical finasteride-minoxidil formulation, maintaining partial improvement in hair density during follow-up.

## Discussion

This report describes a male patient with androgenetic alopecia (AGA) and a suboptimal response to conventional therapies who experienced clinically apparent improvement in hair density and scalp coverage after four months of oral bicalutamide exposure. Before bicalutamide was initiated, the patient had demonstrated only limited clinical improvement despite prolonged treatment with topical minoxidil, oral minoxidil, and oral finasteride.

AGA is characterized by progressive follicular miniaturization driven by androgen signaling in genetically predisposed individuals. Current approved therapies primarily reduce androgenic stimulation of the hair follicle or promote hair growth; however, treatment responses are variable, and some patients continue to experience disease progression despite therapy [1,4]. Bicalutamide is a nonsteroidal selective androgen receptor antagonist that has been investigated as an off-label treatment for female pattern hair loss, where clinical improvement has been reported in selected patients [2,3,5]. In contrast, evidence on its use in male AGA remains extremely limited because of concerns about clinically significant systemic adverse effects [2,3,5-7].

An important aspect of this case is that both topical and oral minoxidil were discontinued before oral bicalutamide was initiated. Therefore, the observed hair regrowth occurred in the absence of concomitant minoxidil therapy, reducing an important potential confounding factor. Nevertheless, because this is a single clinical observation, a causal relationship between bicalutamide exposure and hair regrowth cannot be established. Likewise, a delayed contribution from previous therapies cannot be completely excluded.

The differential clinical response observed after androgen receptor blockade may generate hypotheses regarding the role of receptor-level signaling in AGA. Whereas finasteride inhibits the conversion of testosterone to dihydrotestosterone (DHT), thereby reducing androgenic stimulation of the hair follicle, bicalutamide acts downstream by directly antagonizing the androgen receptor, preventing both testosterone and DHT from activating androgen receptor signaling within the hair follicle [1,4,5]. Although this mechanistic difference may provide additional insight into the biology of androgen signaling in hair follicles, it should not be interpreted as evidence of therapeutic efficacy.

The patient developed gynecomastia, mastodynia, decreased libido, erectile dysfunction, and mild elevations in hepatic enzymes during treatment, findings that are consistent with the recognized adverse effect profile of systemic bicalutamide [5-7]. Mild elevations in liver enzymes have been described during bicalutamide therapy, supporting the importance of baseline and periodic laboratory monitoring [5-7]. Although the patient's clinical adverse effects resolved after treatment discontinuation, follow-up liver function tests and hormonal evaluations were unavailable; therefore, biochemical recovery and endocrine normalization could not be assessed. Furthermore, because alcohol consumption during treatment was not documented and a viral hepatitis panel was not performed, the observed liver enzyme abnormalities cannot be attributed solely to bicalutamide.

This case has several limitations. As a single-patient observation, it cannot establish causality or estimate treatment efficacy. Hormonal measurements before, during, and after treatment were unavailable, precluding assessment of endocrine changes associated with androgen receptor blockade. Follow-up liver function tests after treatment discontinuation were also unavailable, preventing confirmation of biochemical recovery. In addition, alcohol consumption during treatment was not documented, and a viral hepatitis panel was not performed. Therefore, alternative causes of the transient hepatic enzyme elevations, including viral hepatitis, cannot be completely excluded. Finally, treatment response was assessed using serial clinical photographs and routine dermatologic examination rather than standardized quantitative methods such as phototrichogram analysis.

Rather than supporting the use of oral bicalutamide for male AGA, this case documents a temporal association between androgen receptor blockade, clinically apparent hair regrowth, and clinically significant adverse effects. Although this observation may contribute to a better understanding of androgen receptor signaling in AGA, it should be interpreted cautiously given the inherent limitations of a single case report. Further controlled studies are needed to better characterize the biological effects of androgen receptor antagonism, establish its safety profile, and determine whether this therapeutic approach has any role in the management of male AGA.

## Conclusions

This case describes a temporal association between oral bicalutamide exposure and clinically apparent hair regrowth in a male patient with androgenetic alopecia who had demonstrated a suboptimal response to conventional therapies. Although the observed response is biologically plausible given the role of androgen receptor signaling in follicular miniaturization, causality cannot be established because previous therapies and other potential contributing factors may have influenced the clinical outcome.

The findings highlight the potential relevance of direct androgen receptor blockade as a topic for future investigation rather than evidence supporting therapeutic efficacy. At the same time, the occurrence of endocrine, sexual, and hepatic adverse effects underscores the limitations of systemic bicalutamide use in male patients. Carefully designed controlled studies are needed to better define the safety profile, clinical efficacy, and potential role of androgen receptor antagonism in androgenetic alopecia before any conclusions regarding the use of oral bicalutamide in male AGA can be drawn.
